# Multi-Parametric Study on Flexural Behavior of Wool–Flax Hybrid Composites Under Thermal Conditions

**DOI:** 10.3390/ma18143219

**Published:** 2025-07-08

**Authors:** Tsegaye Lemmi, David Ranz, Clara Luna Martin

**Affiliations:** 1 Institute of Architecture of Textiles, Faculty of Material Technologies and Textile Design, Lodz University of Technology, 116 Zeromskiego Str., 90-924 Lodz, Poland; 2 Department of Design Engineering and Manufacturing, University of Zaragoza, C/María de Luna, 50018 Zaragoza, Spain; dranz@unizar.es (D.R.); claraluna.martin@unizar.es (C.L.M.)

**Keywords:** wool, flexural properties, sustainability, biocomposites, thermal stability, flax, curing, hybrid composites

## Abstract

The increasing demand for sustainable materials has intensified the interest in natural fiber-reinforced composites (NFRCs) as environmentally friendly alternatives to synthetic composites. However, NFRCs often face limitations in thermal stability, restricting their use in high-temperature environments. To address this, the present study explores the hybridization of cellulosic flax fibers with protein-based wool fibers to improve thermal stability without compromising mechanical integrity. Wool–flax hybrid composites were fabricated using a bio-based epoxy resin through a resin infusion technique with different fiber proportions. The flexural properties of these composites were evaluated under varying temperature conditions to assess the influence of fiber composition and thermal conditions. This study specifically examined the impact of wool fiber content on the flexural performance of the composites under thermal conditions, including behavior near and above the matrix’s glass transition temperature. The results showed that the flexural properties of the hybrid biocomposites were significantly affected by temperature. Compared with specimens tested at room temperature, the flexural modulus of all variants decreased by 85–94%, while the flexural strength declined by 79–85% at 120 °C, depending on the variant. The composite variant with a higher wool content (variant 3W) exhibited enhanced flexural performance, demonstrating an average of 15% greater flexural strength than other variants at 60 °C and 5% higher at 120 °C. These findings suggest that incorporating wool fibers into flax-based composites can effectively improve thermal stability while maintaining flexural properties, supporting the development of sustainable biocomposites for structural applications.

## 1. Introduction

In recent years, textile-reinforced composite materials have found applications in various industries, including the automotive, aerospace, construction, and marine sectors, where lightweight and high-strength materials are increasingly favored. Most of these composites available on the market for various applications are made from glass or carbon fibers. However, due to the urgent environmental issues related to climate change and pollution, there is a growing need to focus on sustainable alternatives. In light of these environmental challenges, there has been considerable interest in utilizing natural fibers as a composite reinforcement [1,2].

Natural fiber-reinforced composites (NFRCs) have emerged as sustainable alternatives to synthetic fiber-reinforced composites primarily due to their biodegradability, renewability, low cost, recyclability, reduced environmental footprint during production, and disposal phases [3,4,5,6,7]. Flax (*Linum usitatissimum* L.) fiber is one of the most widely used natural fibers for composite reinforcement. Flax fiber is a lignocellulosic fiber, and its physical characteristics are primarily influenced by its main components: cellulose, hemicellulose, and lignin [8]. Among these, cellulose is the stiffest and strongest organic element within the fiber. However, cellulose is a semicrystalline polysaccharide that contains many hydroxyl groups, making natural fibers like flax inherently hydrophilic. This hydrophilic nature leads to a weak bond and poor compatibility when used with hydrophobic matrix materials, resulting in a poor fiber–matrix interface, high moisture absorption, and low thermal stability. The low thermal stability increases the possibility of cellulosic degradation and the possibility of emissions of volatile materials that could adversely affect the composite properties. The low thermal stability limits the use of cellulosic fiber-based composites to low-temperature applications [9,10,11]. The high moisture absorption is also one of the challenges that limit the widespread use of NFRCs. However, many scholars have taken various surface modification approaches to reduce the hydrophilicity of the natural fibers [12,13]. Despite this drawback, flax has gained considerable attention due to its excellent mechanical properties, particularly in terms of tensile and flexural performance, owing to its high cellulose content (approximately 70%) and inherent stiffness [14,15].

Enhancing the thermal stability of NFRCs without compromising their mechanical properties is crucial for expanding the applicability of these composites to structural and semi-structural components. The hybridization of fibers offers a potential route to overcoming individual fiber limitations by leveraging the complementary properties of different fibers. The concept of hybridizing reinforcement materials in composite manufacturing has been advancing in recent years. However, this concept primarily focuses on the hybridization of man-made fibers with natural fibers [16,17,18]. The idea of hybridizing different types of natural fibers is still emerging. In this work, the hybridization of flax and a wool fiber-based reinforcement was explored.

Wool is a keratin-based protein fiber with a highly complex structure. Wool fiber possesses remarkable thermal insulation properties and flame resistance compared to cellulose-based fibers [19,20,21]. Recent advancements in wool–polymer composites have been presented in the literature [22], detailing the mechanical and physical features of these composites and exploring a range of potential applications. Wool fiber-reinforced composites can exhibit a lower coefficient of thermal conductivity, which can prolong the material’s decomposition under thermal conditions. This property positions composite materials reinforced with wool fibers for various applications where thermal insulation is required [23,24]. The integration of wool with flax in a hybrid biocomposite structure could result in a material that combines the high mechanical strength of flax with the thermal stability of wool.

Flexural stress and the flexural modulus are critical properties for evaluating the suitability of composite materials in load-bearing and structural applications. These properties, which include flexural strength and the flexural modulus, are highly dependent on fiber type, fiber orientation, matrix characteristics, and processing conditions, including post-curing treatments [25,26]. Moreover, flexural performance under thermal loading is of significant interest, especially for components exposed to variable service temperatures, as the matrix and fiber interface can be profoundly affected near the glass transition temperature of the polymer matrix.

This study investigates the influence of temperature and material properties on the flexural behavior of hybrid wool–flax fiber-reinforced composites manufactured using a bio-based epoxy resin. The objectives are to understand the role of wool fiber incorporation on the thermal and mechanical response of the composite, analyze how varying wool-to-flax ratios influence flexural performance, and assess the composite’s behavior at temperatures approaching and exceeding the glass transition temperature of the matrix. This investigation aims to contribute to the ongoing development of thermally stable, mechanically robust biocomposites that could serve as sustainable alternatives for conventional synthetic composites in structural applications. The findings from this research will contribute to optimizing NFRCs’ design and improving their applicability in thermally demanding environments such as automotive components, drone parts, structural elements in construction, and outdoor applications. By bridging the knowledge gap in temperature-dependent behavior, this study seeks to enhance the reliability and performance of NFRCs in real-world applications.

## 2. Materials and Methods

### 2.1. Materials

This study utilized wool and flax woven fabric with plain and 2/2 twill weave structures, respectively, as the reinforcing phase, along with a bio-based resin as the matrix phase. The flax fabric with an areal weight of 500 g/m^2^ was sourced from the Libeco company (Meulebeke, Belgium). The wool fabric with an areal weight of 400 g/m^2^ was purchased from Zakłady Przemysłu Wełnianego TOMTEX S.A. (Tomaszów Mazowiecki, Poland). The flax fabric was selected due to its good mechanical properties compared with other natural fiber-based fabrics, sustainability, and lightweight characteristics. The wool fabric was selected primarily due to its inherent thermal insulation, flame resistance, and biodegradability, making it an advantageous choice for applications that require environmental sustainability and temperature control in sensitive environments. For the matrix, a medium-viscosity bio-epoxy resin, SR GreenPoxy 33 supplied by Sicomin Epoxy Systems (Pluguffan, France), was used in combination with its SD 4773 hardener. SR GreenPoxy 33 is an epoxy resin that has 35% of its molecular structure sourced from a plant origin. The green epoxy resin was explicitly selected for its compatibility with natural fibers and low environmental impact.

### 2.2. Composite Preparation

**Figure 1:**
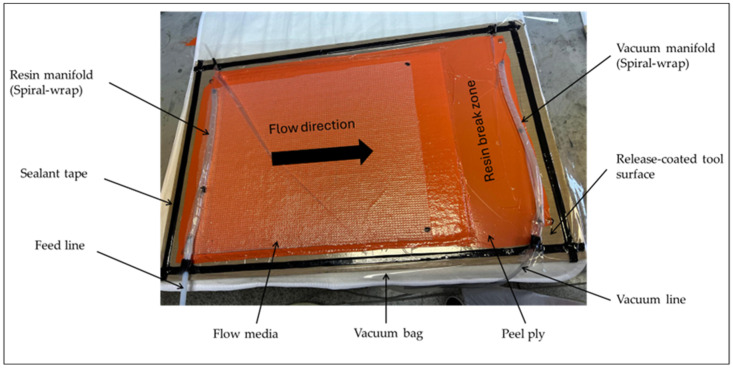
Composite preparation using resin infusion technique.

The composite was produced using a resin infusion technique, shown in Figure 1. The resin infusion process began by arranging the preform layers in the desired orientation on a glass plate. Depending on the arrangement of the preforms, five variants of composite samples were produced. These variants are shown in Table 1. A peel ply and flow mesh were placed over the preform stack to aid resin flow, and the setup was sealed with a vacuum bag. The resin was mixed with the hardener according to the mixing ratio specified by the manufacturer and degassed to remove any air bubbles. After air leak testing, a vacuum pump (Becker Iberica, Vilanova i la Geltru, Spain) created a pressure differential to draw the resin through an inlet port and into the fabric layers. Once infused, the composite was cured under vacuum at room temperature for 24 h. Some specimens from each sample variant were post-cured at 60 °C for 24 h in the laboratory oven (Hobersal, Caldes de Montbui–Barcelona, Spain) to enhance mechanical properties and understand the effect of post-curing on the composite’s properties. The final composite was inspected for uniformity and structural integrity before being subjected to experimental investigations.

This study focused on evaluating the viability of incorporating wool fiber into composite materials due to its inherent thermal stability and flame retardancy. These properties were key considerations in the design of the reinforcement layer arrangement for the variants presented in Table 1. Flax fabric was selected to enhance the mechanical properties of the composite. The primary objective was to develop sustainable composite materials reinforced with natural fibers, achieving improved thermal and mechanical performance without the need for additional flame-retardant treatments. To assess the effect of wool incorporation, a composite sample reinforced with four layers of wool fabric was initially produced to evaluate its mechanical performance. Similarly, a composite with four layers of flax fabric was prepared to analyze the mechanical behavior of flax-reinforced composites. Following this, three hybrid composite variants were designed with wool-to-flax layer ratios of 1:1, 2:1, and 3:1, as outlined in Table 1. In these hybrid composites, flax layers were positioned in the central layer of the composite structure to contribute to mechanical properties, while wool layers were arranged on the outer surfaces to enhance the thermal insulation and fire resistance of the composite materials.

To investigate the effect of temperature on the mechanical performance, specifically, the flexural properties of the composite samples, experiments were conducted at three designated temperatures: room temperature (20 °C), 60 °C, and 120 °C. These temperatures were selected based on the thermal behavior of flax and wool fibers, as well as the glass transition temperature (Tg) of the matrix material used for composite fabrication (87 °C to 96 °C). The testing temperatures of 20 °C, 60 °C, and 120 °C were selected to assess the flexural behavior of the composite at room temperature, below, and above the Tg of the matrix, respectively. Testing the composites at temperatures higher than the resin’s Tg aimed to explore the flexural properties and thermal limits of the hybrid composite under extreme conditions. This can be relevant in specific scenarios, such as exposure to elevated temperatures or overheating. Evaluating the composite’s performance above its glass transition temperature (Tg) helps define the operational boundaries and failure modes, providing a more comprehensive understanding of the composite’s thermal stability.

The specimen representation was coded based on the variant type, the testing direction relative to the warp and weft yarns arrangement in the composite structure, the curing process, and the testing temperature. Specimens were labeled using the format pZx-y, where

p represents the post-curing process,
Z denotes the variant type,
x indicates the specimen testing direction,
y corresponds to the testing temperature.

For instance, the specimen labeled as pW0-120 indicates that the specimen underwent the post-curing process (p) and was from sample variant W, as listed in Table 1. The number 0 indicates that the specimen was tested in the warp direction at 120 °C.

Table 2 provides a detailed representation of specimens of composite variant W (as listed in Table 1). Specimens for the other composite variants listed in Table 1 follow the same coding format. In Table 2, the symbol Θ indicates that the sample underwent the specified process.

### 2.3. Experimental Investigation

**Figure 2:**
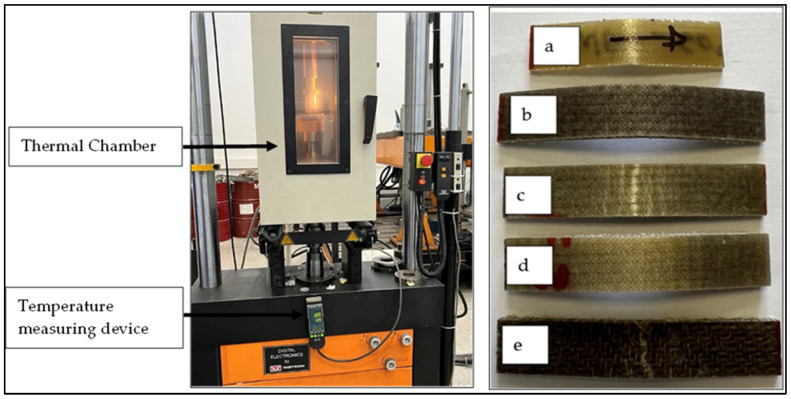
Instron machine with a thermal chamber, and post-curing specimens after flexural tests at 60 °C. (**a**) W, (**b**) 1W, (**c**) 2W, (**d**) 3W, (**e**) F.

An extensive flexural test campaign was conducted on the fabricated composite sample variants. The experiment was conducted to assess the impact of wool layers on the reinforcement structure and the effect of temperature on the flexural properties of the composite materials. The three-point flexural test of the composite samples was conducted using a servo hydraulic universal Instron machine model 8032 (Instron, Norwood, MA, USA), connected to a heating chamber, as shown in Figure 2. The machine has a 5 kN load cell and a loading rate of 2.5 mm/min. The machine was calibrated to ensure the credibility of the tests. The specimens were preheated in the thermal chamber mounted to the Instron machine at the designed testing temperature for the samples. The specimens were prepared for testing according to the ISO 14125 test standard [27], depending on the thickness of the composite variants. The average results of five specimens for each variant were recorded for flexural strength and flexural modulus, both in warp and weft arrangements of the fabrics in the composite structure.

## 3. Results and Discussion

This section presents and discusses the results of the experiments conducted on all the composite variants considered in this study. Stress–strain curves and bar graphs illustrating the flexural modulus and flexural stress of the composite samples are analyzed from multiple perspectives. The results are discussed under four distinct comparative frameworks.

Firstly, an analysis was conducted to examine how the flexural properties vary with changes in material composition. Secondly, the influence of fiber orientation on the flexural behavior of the composite samples was evaluated. Thirdly, a comparative analysis of post-cured and non-post-cured specimens was conducted to identify any significant impact of the curing process on the flexural properties of the composite materials. Finally, the effect of temperature on the flexural properties of the composite samples was investigated.

### 3.1. Material Influence

**Figure 3:**
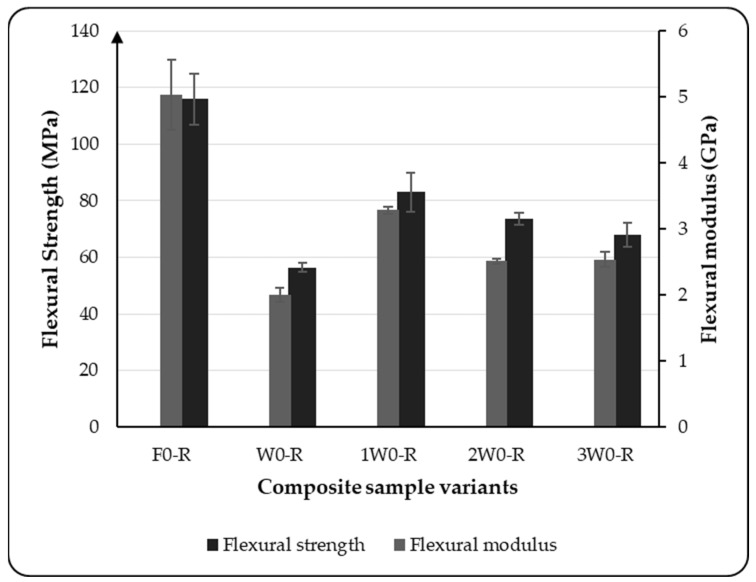
Flexural properties of all composite variants tested at room temperature.

**Figure 4:**
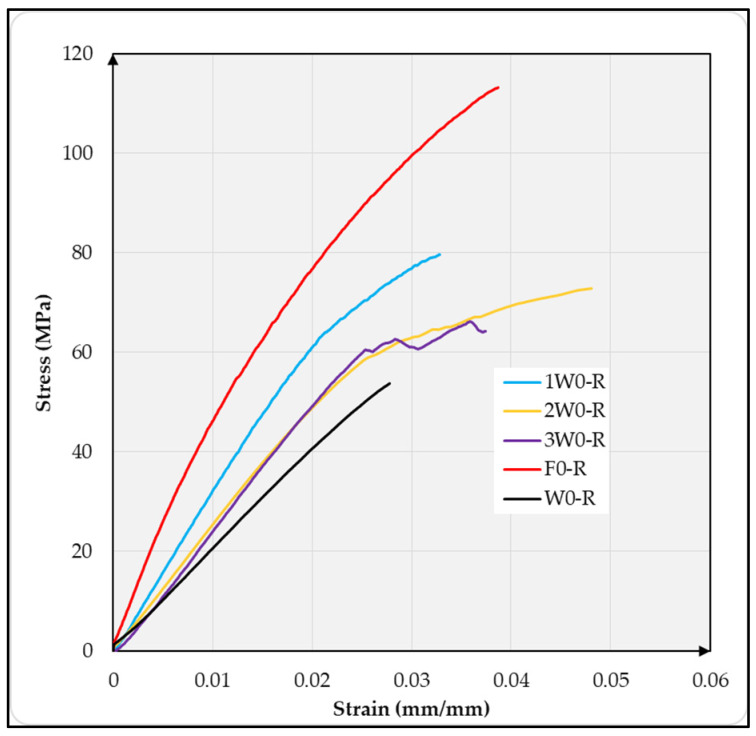
Stress–strain curve of all composite variants tested at room temperature.

The flexural results obtained for the composite variants considered in this study are presented in Figure 3 and Figure 4. The bar graph in Figure 3 illustrates the flexural strength and flexural modulus of the five composite variants tested in the warp direction of the fabric arrangement in the composite. The results indicate that the composite sample composed entirely of flax, as well as those with a higher proportion of flax, exhibited greater flexural strength and modulus compared with those dominated by wool, as shown in Figure 3. The results show that the flexural modulus and flexural strength of the composite variants decreased as the proportion of wool layers in the reinforcement increased. However, compared with the composite variant reinforced only with wool fabric, the modulus increased by 48.21%, 32.14%, and 21.43% for the 1W, 2W, and 3W variants, respectively. This indicates that the flexural modulus of wool-based composites is lower than that of flax-reinforced composites. This is due to the fact that wool fiber contains keratin, a flexible protein with a coiled structure that is prone to bending, whereas flax fiber is composed of cellulose, which is a rigid, crystalline structure. Similarly, the flexural strength was also dependent on the proportion of the flax fabric present in the composite reinforcement. The higher the proportion of flax fabric in the composite reinforcement, the higher the flexural strength of the composite. This behavior can be attributed to the inherent strength of flax, which has a more significant influence on the mechanical properties of the composite materials. As presented in Figure 3, the flexural properties of the variant F0-R were observed to be quite different compared with the other variants, which evolved from wool fabric. This is a result of flax fiber’s structure; flax is known for its high stiffness along the fiber axis due to the crystalline cellulose in its fiber structure. However, flax fibers are relatively brittle and more prone to microcracking under stress, which can limit the flexural strength. The previous study [28] conducted on a flax-reinforced composite also shows the brittleness and microcracking that have an influence on the flexural properties of the composite.

As shown in Figure 4, the stress–strain behavior of the composite variants studied reveals a considerable difference between the flax-reinforced composite variants and those produced from wool and the wool–flax hybrid. As shown in the graph, the wool-only reinforced variant exhibited the most deformation. The variants with hybrid fabric showed less deformation compared with the entirely wool fabric-reinforced composites. The composites made entirely from flax fabric exhibited the least overall deformation. This phenomenon was also due to the fiber structure of flax and wool fibers.

### 3.2. Influence of Fiber Orientation

**Figure 5:**
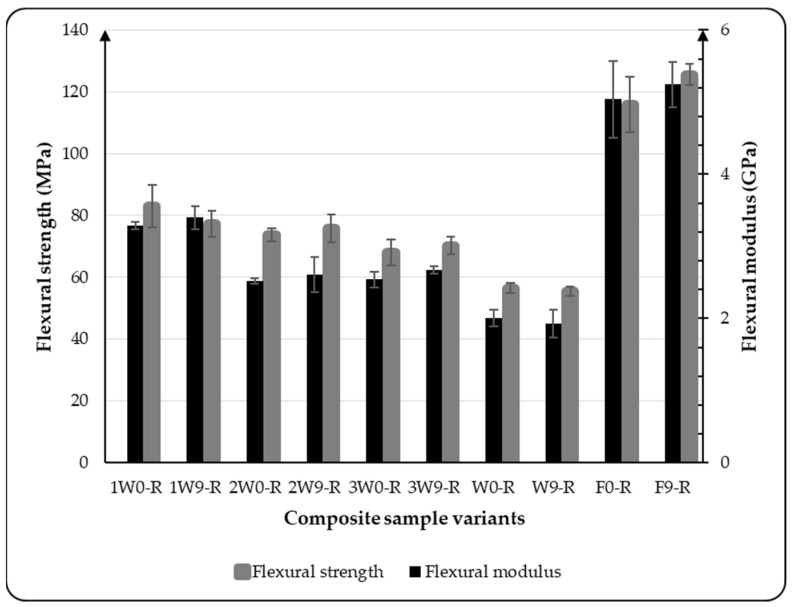
Flexural properties of composite variants tested at room temperature in different orientations.

Fiber orientation plays a critical role in determining the mechanical properties of fiber-reinforced composites. The alignment of fibers in the composite structure affects how loads are carried and distributed throughout the material. Previous research has shown that fiber orientation influences the flexural strength and flexural stiffness of composite materials [29,30]. In Figure 5, the impact of fiber orientation on the flexural strength and flexural modulus of composite variants tested parallel (0°) and perpendicular (90°) to the fiber direction is presented. The results do not show any significant differences based on the testing direction. This is believed to be due to the use of woven fabric as the reinforcement, where fibers are arranged in both 0° and 90° directions. As a result, the composite exhibits a balanced flexural behavior.

### 3.3. Post-Curing Effects

**Figure 6:**
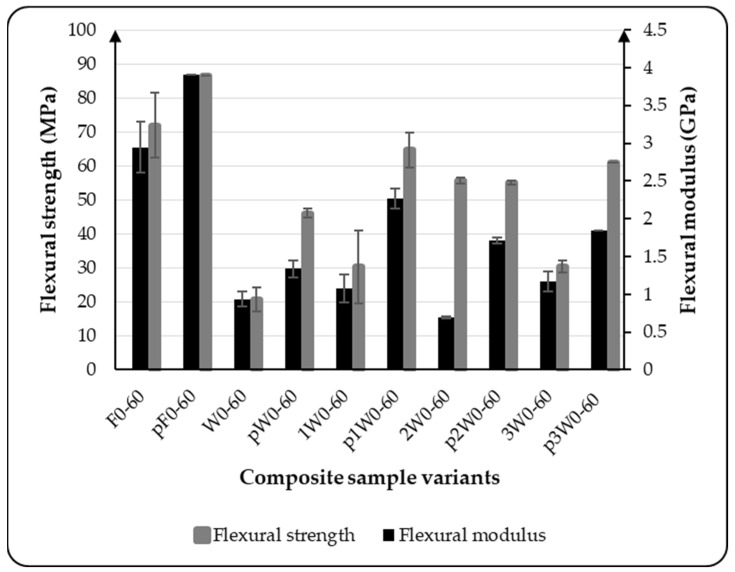
Influence of post-curing process on the flexural properties of composite samples.

Post-curing is a thermal treatment applied to composite materials following an initial cure, which was conducted at room temperature in this study. This process significantly affects the mechanical performance of thermoset composites. In this study, the composite samples were post-cured at a temperature of 60 °C for 24 h prior to flexural testing. The post-curing temperature was selected based on the thermal properties of the matrix material. Figure 6 presents the effect of post-curing on each composite variant. The bar graph compares the flexural strength and flexural modulus of samples tested before and after post-curing. The flexural tests of both cured and non-post-cured samples were conducted at a temperature of 60 °C to ensure consistency in testing conditions.

The flexural results obtained indicate a significant increase in both the flexural strength and flexural modulus for all composite variants considered in this study, regardless of the type of reinforcement used. Post-curing leads to a modification of the mechanical properties of the composites [30]. The flexural strength of the samples increased by 32.65%, 44.09%, 110.19%, 144.29%, and 58.12% for variants F, W, 1W, 2W, and 3W, respectively, after the post-curing process. As shown in the bar graph, the flexural modulus of these composites also increased substantially following post-curing. These results signify that the thermal treatment, applied after the initial cure, promoted additional crosslinking within the polymer matrix, resulting in a denser network structure. As a result, post-cured composites typically exhibit improved flexural strength and stiffness due to enhanced load transfer efficiency between the matrix and the reinforcing fibers.

Furthermore, post-curing contributes to better interfacial adhesion and reduces the residual stresses developed during the primary curing stage, which may otherwise compromise mechanical integrity. The process also enhances thermal stability, allowing the composite to maintain its flexural performance at a testing temperature of 60 °C. However, improper post-curing conditions, such as excessive temperature or duration, can lead to embrittlement or the thermal degradation of the matrix as well as the fiber. Therefore, optimizing post-curing parameters is essential to fully realize the mechanical benefits without compromising the material’s durability.

### 3.4. Temperature Effect

One of the primary drawbacks of natural fiber-reinforced composites is their limited thermal stability. Numerous studies have explored various approaches to address this limitation [31,32,33,34]. In this study, a hybridization strategy was adopted by combining wool fiber, known for its inherent thermal stability due to its unique chemical composition and physical structure, with cellulosic flax fiber, which has relatively low thermal stability.

**Figure 7:**
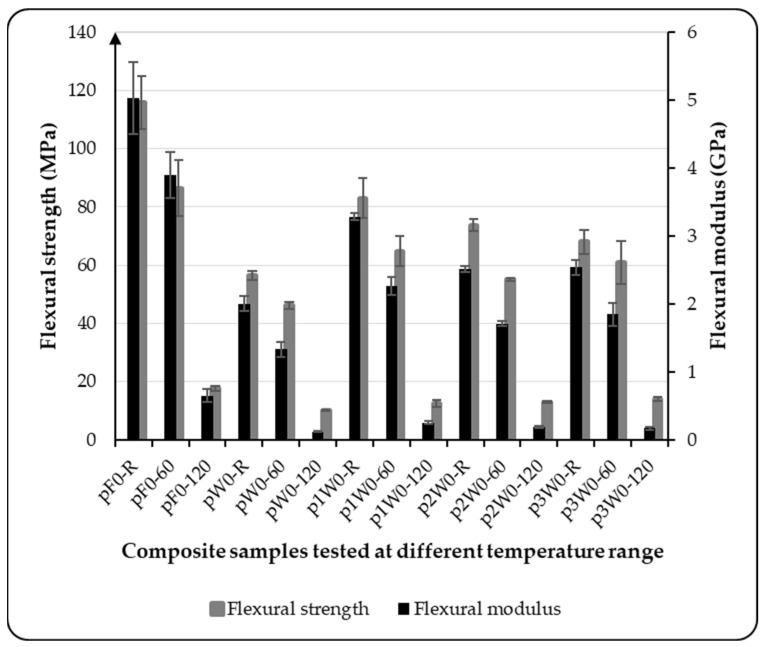
Effect of temperature on the flexural properties of biocomposites.

To evaluate the flexural performance of the composite variants, the samples were subjected to flexural testing at room temperature, 60 °C, and 120 °C. This allowed assessment of the influence of temperature on the composites’ flexural properties and analysis of the benefits of increasing the proportion of wool fiber reinforcement in the composite structure. The results, presented in Figure 7, clearly show that the flexural properties of the biocomposites are significantly affected by temperature. Compared with the specimens tested at room temperature, the flexural modulus of all variants decreased by 85% - 94%, while the flexural strength declined by 79% - 85% at 120 °C, depending on the variant. These reductions are closely linked to the performance of the bio-epoxy resin, the matrix material used in the composites. The testing temperature of 120 °C exceeded the glass transition temperature (Tg) of the matrix, which ranges from 87 °C to 96 °C depending on the curing conditions, as reported by the manufacturer. Testing above the Tg significantly reduces the matrix stiffness, leading to a drastic drop in flexural modulus. Additionally, the elevated temperature weakens the interfacial bonding between the matrix and the reinforcement, leading to deterioration in flexural strength.

**Figure 8:**
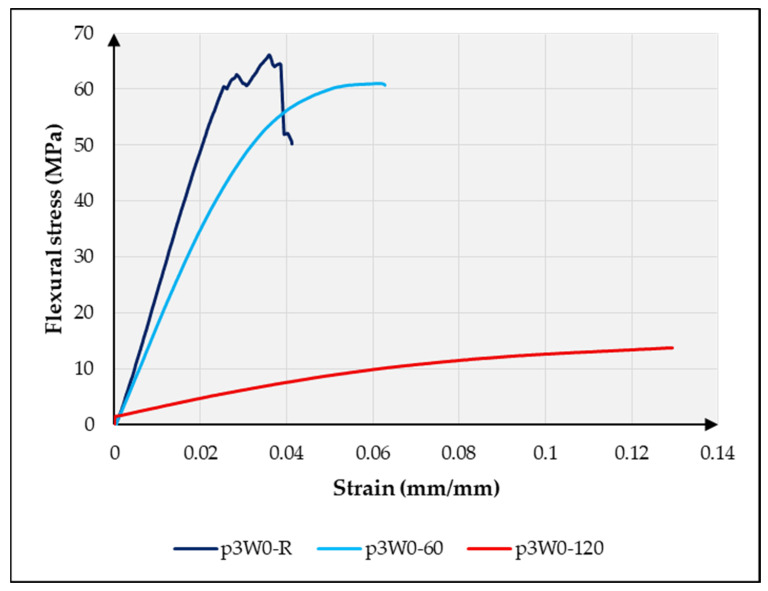
Effect of temperature on the flexural stress–strain curve of composite variant 3W.

In the case of the specimens tested at a temperature of 60 °C, a substantial decrease in the flexural properties was also observed compared with the specimens tested at room temperature. The flexural strength of the pure flax sample decreased by only 25.39% when tested at 60 °C compared with the sample tested at room temperature, as shown in Figure 7. This indicates that the pure flax laminates exhibited relatively higher mechanical properties compared with the variants with a lower wool content, 1W and 2W. However, the sample with a higher percentage of wool performed better, with a flexural strength reduction of only 10.28% under the same conditions. However, increasing the number of wool layers in the reinforcement improved the thermal stability of the composite. The flexural strength of the composite variant 3W was, on average, 15% higher than that of the variants with a lower percentage of wool at 60 °C and 5% higher at 120 °C. The effect of the temperature on the stress–strain curve is shown in Figure 8 for composite variant 3W. These results indicate that incorporating a higher proportion of wool fiber-based preforms in the composite reinforcement enhances its thermal stability to some extent. The thermal stability of wool fiber is due to the presence of keratin protein in its structure. Keratins are extremely strong structural proteins characterized by high thermal stability and low solubility. Keratins are classified into α- and β-types. The α-keratin proteins are organized as coiled coils, and wool is typically considered a representative of α-keratin. The keratin structure of wool contributes to its thermal resistance through a tightly crosslinked, hierarchical protein arrangement, primarily composed of α-keratin. At the molecular level, this structure is stabilized by strong disulfide bonds formed through crosslinking bridges between cysteine amino acid residues, which resist thermal degradation and maintain fiber integrity at elevated temperatures [35,36]. Additionally, hydrogen bonds and hydrophobic interactions within the α-helical structure provide secondary stabilization, limiting molecular mobility as heat increases. Collectively, these molecular features make wool inherently resistant to high temperatures without compromising its structural integrity.

## 4. Conclusions

This study investigated the flexural properties of biocomposites under varying temperatures and examined the effect of incorporating wool fibers into the reinforcement to enhance the thermal stability of the composite. Flax and wool fabrics were combined in a composite structure with a bio-based epoxy resin. This study thoroughly analyzed the influence of different reinforcement materials, fiber orientation, post-curing processes, and thermal conditions on the flexural behavior of the composites. Flexural properties are critical mechanical characteristics for the structural applications of composites. This work aimed to evaluate the flexural properties of biocomposites by combining natural fibers with distinct characteristics. The key findings from this investigation are as follows:

- In flax–wool hybrid composites, increasing the wool content results in a reduction in the composite’s flexural properties.
- Post-curing at 60 °C for 24 h significantly enhances both the flexural modulus and flexural strength of the biocomposites, regardless of the reinforcement type.
- The flexural properties deteriorate when the composite is exposed to temperatures above the glass transition temperature of the matrix.
- A higher proportion of wool in the reinforcement improves the thermal stability of the composites, while flax fibers enhance the flexural performance of the composites.

Overall, this study highlights the potential of addressing the thermal stability limitations of cellulose-based natural fiber composites by incorporating keratin-based wool fibers as a reinforcement material. However, the flexural performance of biocomposites under thermal conditions remains critically dependent on the glass transition temperature of the matrix. Hybrid wool–flax biocomposites have significant potential for lightweight structural component applications, including drone frames, interior automotive panels, sporting goods, building wall panels, and electronic device casings. This study is limited to a specific type of natural fiber hybrid (wool–flax) and resin type, and it examined the flexural behavior of these hybrid biocomposites under thermal conditions. While this provides valuable insight into the thermo-mechanical performance of these biocomposites, the results may not be directly generalized to other natural fiber combinations.

## Figures and Tables

**Table 1:** Reinforcement arrangement of the composite sample variants.

Composite Variants Representation	Reinforcement Arrangement	Matrix
W	Wool-Wool-Wool-Wool	
F	Flax-Flax-Flax-Flax	
1W	Wool-Flax-Flax-Wool	Bio-Epoxy resin
2W	Wool-Wool-Flax-Flax-Wool-Wool	
3W	Wool-Wool-Wool-Flax-Flax-Wool-Wool-Wool	

**Table 2:** Description of specimen coding for variant W (wool-wool-wool-wool).

Composite Samples Representation	Curing Temperature	Specimen Testing Direction		Testing Temperature		
	60 °C	Warp 0°	Weft 90°	20 °C (R)	60 °C	120 °C
W0-R	-	Θ	-	Θ	-	-
W9-R	-	-	Θ	Θ	-	-
W0-60	-	Θ	-	-	Θ	-
W9-60	-	-	Θ	-	Θ	-
pW0-60	Θ	Θ	-	-	Θ	-
pW0-120	Θ	Θ	-	-	-	Θ

## Data Availability

The original contributions presented in this study are included in the article. Further inquiries can be directed to the corresponding author.
